# Supplementary material for: Use of tobacco during COVID-19: A qualitative study among medically underserved individuals
Source: PLoS One. 2024 Aug 19;19(8):e0308966. doi: 10.1371/journal.pone.0308966 (PMC11332913; doi:10.1371/journal.pone.0308966)
Supplement: S1 File — (DOCX) [file pone.0308966.s001.docx]

**Patients Interview Guide**

Last Updated: February 1, 2021

*AIM 1: Impact of COVID-19 on Tobacco Use and Preparedness for Smoking Cessation*

Interviewer focus areas

Impact of COVID-19 on:

- Smoking behavior
- Ease of getting cigarettes
- Management of chronic health conditions/relationship to smoking behaviors
- Attitudes about quitting before/during/after (now)

Prompt*:* We are going to begin the interview now. The COVID-19 pandemic is affecting peoples’ lives in different ways. We are interested to hear how COVID might have affected how you feel about your health.

1. **Can you please tell me about your health?**
   - General health status
   - Chronic conditions/illnesses
     - *i.e. diabetes, cholesterol, high blood pressure, asthma, COPD, HIV*
   - What was the status of your health before the pandemic started?
2. **Can you please describe a normal day in your life (routine) before the pandemic.**
   - Please describe what your smoking was like before the pandemic?
   - How long after waking up do you have to smoke your first cigarette?
   - What benefits do you feel when you smoke?
   - Before the pandemic, how many cigarettes were you smoking per day?
3. **Can you describe how your life has changed since the pandemic began?**
   - Can you please describe if there’s been a change in your smoking since the pandemic began, if any?
   - Since the pandemic began, how many cigarettes do you smoke per day?
   - How has your financial situation changed since the pandemic began?
     - (i.e. changes in employment)
4. **Walk me through how you got cigarettes before the pandemic.** (i.e. places you would go to buy cigarettes*)*
   - Before the pandemic, describe how your finances/economic possibilities (income) were affected by buying cigarettes?
   - Before the pandemic, please describe what places would you go to buy cigarettes?
     - Transportation?
   - Before the pandemic, who provided you with cigarettes?
   - Before the pandemic, how many cigarette packs did you buy at a time?

[The following questions are related to during the pandemic that is from mid-March to now.]

1. **Restrictions were stricter from March to June. During those months, how did you get cigarettes?**
   - From March to June, how did you get cigarettes?
     - Were you hesitant to go out for cigarettes?
   - From March to June, where did you get cigarettes? What places?
   - From March to June, who provided you with cigarettes?
   - From March to now, have your finances been affected by buying cigarettes?
2. **Restrictions have loosened since June to now. How do you get cigarettes now?**
   - Since June to now, describe if you have had any difficulty getting cigarettes?
   - Since the pandemic began, how many packs of cigarettes do you buy at a time?
3. **Can you describe what you have heard about the link between COVID-19 and smoking?**
   - I know you’ve talked about this a little already: Please describe, how, if at all, has the COVID pandemic changed your smoking?
     - In what ways?(*ex. have more time, time alone*)
   - Describe how COVID has affected:
     - How often you smoke?
     - How often you buy cigarettes?
     - Where you buy cigarettes?
   - [This question is more of a “what if” scenario] If you were to get COVID, please describe how sick do you think you would get?
     - How do you think smoking affects how sick you would get?
4. **Have you ever thought about quitting or smoking less?**
   - Have you ever tried to quit or smoke less?
     - Even for a couple of days/weeks/months?
   - Before the pandemic, what made you want to quit or smoke less?
   - Have you had a conversation with your doctor about quitting or smoking less?
     - Have they prescribed any medications to quit smoking?

- If so, did you use them? Did you like those medications?

- - Since the pandemic began, please describe if your thinking about quitting or smoking less has changed?
    - (*if they* ***want*** *to quit smoking*): What resources would be helpful for you to quit smoking?

- Have you heard of (*1 at a time:* patches, gum, lozenges, quit line, individual counseling, prescribed medications) to stop smoking?

- - - (*if they* ***don’t*** *want to quit smoking*): Can you tell me more about your decision to keep smoking?
    - What benefits do you feel when you smoke?
    - What resources would be helpful to you to incentivize you to stop smoking?
    - Have you heard of (*1 at a time:* patches, gum, lozenges, quit line, individual counseling, prescribed medications) to stop smoking?

1. **Are there any additional thoughts or comments you’d like to share with me today?**

Thank you very much for your time today. Your answers will really help our research project. Please feel free to contact us at [HealthyLungs@pennmedicine.upenn.edu](mailto:HealthyLungs@pennmedicine.upenn.edu) or at 1-844-999-1357 if you have any additional thoughts.

1. **May I please have the best mailing address to send you the $50 for your participation?**
   1. The card will arrive empty, please call us back (484-534-8877: Jannie’s google voice) and let us know you got the card and someone on my team will fill the card with the $50 and will be ready in 10 minutes from that for you to use.
2. **Is there a loved one in your life that** helps with: dressing, bathing, getting around the home or community or eating, taking medicine or talking to doctors and nurses, keeping you company/providing emotional support, doing shopping, managing paperwork such as bills, taking care of chores and meal preparation?
   1. Or involved in your healthcare that would like to participate?
3. **Would you like to participate in other interviews for this study? You will be sent $50 for each interview you complete.** If so, please don’t throw out the card we will be sending.
4. *If the patient mentioned wanting to quit smoking, share* [*smoking cessation resources*](file:///\\pmacs.upenn.edu\depts\BE-4104-CCEB\projects\halpern%20scott\PCORI_Enha\Project%20Management\Trial%20Implementation\Interview%20Guides\Patients\Interviewing%20resources\Distress%20hotline_20201214.docx)*.*

______________________________________________________________________________
